# Experimental and analytical validation of a modular acetabular prosthesis in total hip arthroplasty

**DOI:** 10.1186/1749-799X-2-7

**Published:** 2007-05-16

**Authors:** Francisco Romero, Farid Amirouche, Luke Aram, Mark H Gonzalez

**Affiliations:** 1 Department of Mechanical Engineering, Biomechanics Laboratory, University of Illinois at Chicago, 842 W. Taylor Street, Room 1039, Chicago, Illinois 60607, USA; 2 Department of Orthopaedic Surgery, University of Illinois at Chicago, 1801 W. Taylor Street, Chicago, Illinois, USA

## Abstract

A finite element model has been developed to predict in vivo micro motion between a modular acetabular cup and liner after cement less total hip arthroplasty. The purpose of this study is to experimentally validate the model. Six LVDT sensors were used to monitor the micromotion of the liner when subjected to loading conditions ranging from 250 N to 5000 N. Deformations at points of interest for both the experiment and FEM were compared. Results of the FEM with different coefficient of friction between the liner and the cup were investigated to correlate with the experimental results.

## Background

Polyethylene wear particles generated from the acetabular liner of an acetabular cup are a cause of the osteolysis associated with acetabular cup failure. Wear generation comes from the articulation of the femoral head and the liner and from the articulation of the liner and the metal shell. Wear generated by micromotion at the liner metal shell interface, termed backside wear, is potentially more damaging because of the egress of the particles to the cup bone interface through the screw holes and the central fenestration in the metal shell. These particles then create an inflammatory reaction that can produce osteolysis of the bone, leading to degradation of the interface and eventual failure. The etiology of micromotion between the metal shell and polyethylene is multifactorial. Important factors include the contact stress [10], relative sliding distances [11], conformity between contacting surfaces and the number of screw holes in the acetabular shell [12]. A rigid locking mechanism is paramount in limiting micromotion at the liner shell interface. Conformity of the liner shell interface and polishing of the metallic shell are new advances to limit fractional wear.

The loading of the hip joint is cyclical and occurs during gait. Impact loading of the hip joint can also occur as a result of subluxation and relocation of the metallic ball in the polyethylene cup. Little work has been done on the effect of physiologic loads on micromotion at the cup liner interface. The load patterns are particularly complex during subluxation relocation because the initial contact at relocation may be eccentric creating a moment. The cylical pattern of loading associated with level gait, stair climbing and the impact from eccentric subluxation and relocation are virtually impossible to reproduce physically in the laboratory. To this end a finite element model has been created to investigate micromotion of the metal shell liner interface. The purpose of this experiment is to validate this model. The validation of the model quantifies micromotion of a loaded acetabular cup liner assembly with linear variable differential transducer (LVDT) sensors.

## Methods

### 2.1. Experiment set-up

**Figure 1:**
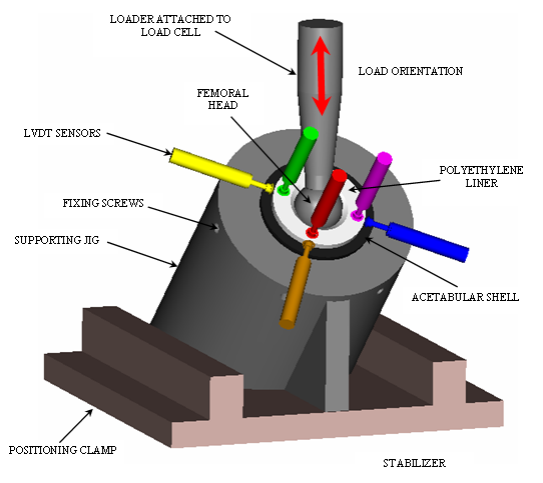
Experiment set-up. Orientation of the acetabular components (acetabular shell, liner and femoral head) with respect to applied load.

Seven commercially available UHMWPE liners, 28 mm inner diameter and 54 mm outer diameter, were used. The experiment set-up is represented in Figure 1.

The acetabular cup was placed into the acetabular shell as recommended by the manufacturer. The acetabular cup was then placed into a custom made jig that held the cup rigidly. (Figure 1 supporting jig). Six circumferential set screws were tightened rigidly to the acetabular cup within the jig. The supporting jig was designed to hold the acetabular shell at a constant angle of 45° with respect to the horizontal simulating a 45° inclination angle. For this experiment the anteversion angle was fixed at 15–20°. The acetabular cup is statically placed at 25 degrees of anteversion

**Figure 2:**
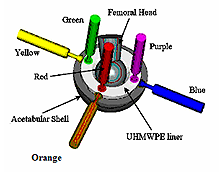
Modular Acetabular components set-up and LVDT sensors' positioning.

Two acetabular shells (66 mm outer diameter) were used during all the experiments. The first had only a polar fenestration and the second had polar fenestration and three screw holes. A sensor positioning plate containing six calibrated LVDT sensors (AC-DC LVDTS 332, TRANS-TEK Inc, Ellington, CT) was rigidly attached to the jig. The LVDT sensors detect linear micromotion in the x, y and z direction. The metal tip of each LVDT sensor was set to zero position when it came in contact with the liner before any loading conditions were applied. Care was taken to locate the sensors' tips away from the liners' anti-rotation tabs. Three of the sensors contacted the liner perpendicularly to the outer flat surface, while the other three contacted the liner perpendicularly from the side (Figure 2). The displacements measured using the six LVDT sensors correspond to the local deformation of the polyethylene liner at the points of contact.

The jig sensor assembly was positioned in an INSTRON machine (Model 5579, Instron Corp., Canton, MA) for loading. Load was applied to the acetabular components through a 28 mm, commercially available femoral head attached to the movable crosshead of the INSTRON machine. The femoral head was positioned in the acetabular liner and adjusted until a conforming contact was achieved without applying significant load. The readings on the sensors were still showing zero displacements. This served as the starting point for experimental measurements of load and micromotion.

The loading characteristics of each trial were set with the INSTRON computer controller (Merlin 5500 Series). The loading characteristics applied to the liner (including number of cycles, speed, and maximum load) were specified. Each specimen (liner) was subjected to the loading profiles described in Table 1.

**Table 1:** Loading profile applied to each liner specimen.

*Loading case*	*Speed (mm/min)*	*Cycles*	*Maximum Load (N)*
1	5	20	250
2	5	20	350
3	5	20	450
4	5	20	550
5	5	20	650
6	5	20	750
7	5	20	850
8	5	20	950
9	5	20	1050
10	5	20	1150
11	5	20	1250
12	5	20	1350
13	5	20	1450
14	5	20	1550
15	5	20	1650
16	5	20	1750
17	5	20	1850
18	5	20	1950
19	5	20	2050
20	5	20	2150
21	5	20	2250
22	5	20	2350
23	5	20	2450
24	5	20	2550
25	10	25	2650
26	10	25	2750
27	10	25	2850
28	10	25	2950
29	10	25	3050
30	10	25	3150
31	10	25	3250
32	10	25	3350
33	10	25	3500
34	10	25	3750
35	10	25	4000
36	10	25	4250
37	10	25	4500
38	10	25	4750
39	10	25	5000
40	10	25	6000
41	0.5	2	10000

TOTAL		882	

In each loading case the load range applied goes from 0 N to the maximum load specified for each loading case.

The LVDT sensors were connected through a Data Acquisition Card (DAQCard-A1-16E-4, National Instruments, Austin, TX) to a laptop computer. Using data analysis software (Lab-View, National Instruments, Austin, TX), a program was developed to transform the signals from the LVDT sensors into micromotion displacement values. The micromotion values from the LVDT sensors were synchronized with the loads applied with the INSTRON machine.

The FEM was then used to generate values for micromotion at the points of attachment of the LVDT on the polyethylene liner. The comparison of results from the experimental and computational models allowed us to validate the finite element model of the modular acetabular component.

### 2.2. Modular acetabular component: CAD model

Three-dimensional computer models of the modular prosthetic components (femoral head, acetabular liner and acetabular shell) were developed using Pro/Engineer 2000i2 (Parametric Technologies Inc., Needham, MA). Component dimensions used in the models were based on precise measurements taken from commercially available products (DePuy, Inc., Warsaw, IN) using hand instruments.

The model was created with a polar fenestration. The same consideration was made to model the liner. Geometrical details without structural significance were omitted in order to import CAD models to the standard ANSYS format (IGES). All 3-D models were converted to IGES format (No-defeaturing option).

### 2.3. Finite Element model

**Figure 3:**
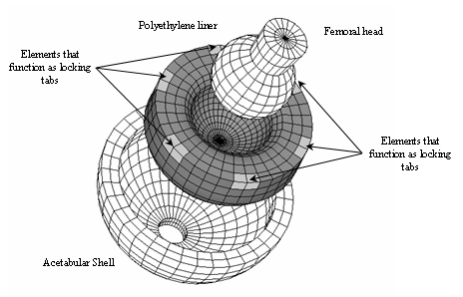
Finite Element Model of the acetabular shell, liner and femoral head. The liner locking mechanism was simulated constraining all degrees of freedom of the nodes located at the same positions as locking tabs of the real-life liner.

A three dimensional 3595 elements FE model (Figure 3) of the femoral head, liner, and acetabular shell was developed based on the CAD geometry previously created using ANSYS/LS-DYNA. The diameter of the articulating surface femoral head/liner measured 28 mm while the liner backside diameter measured 54 mm. A 12 mm diameter polar fenestration was placed in the metal acetabular shell which corresponds to the central insertion hole. The acetabular shell was positioned with 45° inclination and 25° anteversion [13].

**Figure 4:**
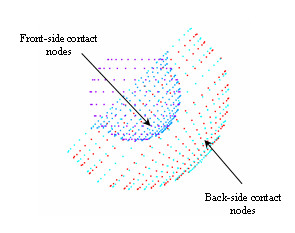
Contacting areas (Acetabular Shell/liner and Liner/femoral head) involved in the Finite Element Model.

The Finite Element mesh of the acetabular shell, liner and femoral head consisted of eight-node hexahedron solid elements (Table 2). A polar mesh design was selected to avoid the nodal penetration problem (Figure 4). The mesh pattern was kept the same in all the contacting areas (outer surface of the femoral head-liner front side and liner back-side and acetabular shell) so that, the nodes placed in the contacting surfaces do not interfere with the target area. A cross section of the liner, acetabular shell and femoral head were first meshed due to axial symmetric conditions of the model. Planar elements were then used having perfect control of the shape of the elements. The planar elements were revolved with a specific angle creating a polar mesh design. The angle of revolution was chosen to avoid singularities that can occur when hexahedron elements are converted to tetrahedron elements [14].

**Table 2:** Material Properties

Component	N° element	FEM mat.	Actual mat.
Femoral Head	501	Rigid	Cobalt-Chrome
Liner	1799	E = 975 MPa ν = 0.46 ρ = 960 Kg/m^3^	UHMWPE
Acetabular Shell	720	Rigid	Titanium

Comparison between the material properties Finite Element model and real-life components material properties.

A linear isotropic material was chosen to model the UHMWPE liner for the proposed experiment. For a study of wear and fatigue a non linear material will be more appropriate. The material properties of the liner were based on the information obtained in the available literature [15], where the Young's Modulus = 975 MPa, and Poisson's Ratio = 0.46. A rigid material was chosen for the acetabular shell and the femoral head components

A linear contact between implant components was modeled using an automatic surface-to-surface contact (ASTS). The acetabular shell was fully constrained, assuming a rigid union between the acetabular shell and the acetabulum. The femoral head was constrained with respect to the rotational degrees of freedom. Perfect conformity between femoral head and liner as well as between liner and acetabular shell was assumed. Anti-rotation features such as the equatorial locking tabs provide the greatest restraint to keep the liner in the proper position within the shell [16]. When load is applied to a modular acetabular component with locking constraints, the liner locking tabs share the greater part of the applied load. In order to simulate the six locking equatorial tabs of the liner, a total of twelve nodes of the liner FE model were fully constrained (Figure 3).

### 2.4. FE validation

**Figure 5:**
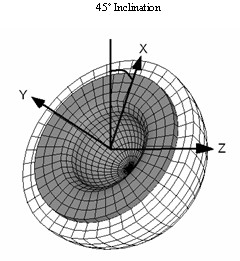
Coordinate system used as a reference for the loads in the FE model. The CS is shown with respect to the acetabular shell inclination angle.

A similar loading profile to the one used in the experiment was created in ANSYS/LS-DYNA in order to validate the experimental results in terms of stability/micromotion of the liner. The load was applied at the center of the femoral head and varied from no load up to 1775 N applied linearly. The maximum load was based on previous studies in gait [17-21] where the hip joint forces were evaluated. In order to reproduce the experimental conditions as closely as possible both the orientation of the applied load (vertical) and the position of the acetabular component (45° inclination) were the same in the computer model and the experiment set-up. (Figure 5)

Published reports of the friction coefficient between the metal and polyethylene ranged from μ = (0.083 to 0.2). [[15],15a] The finite element model was run with friction coefficient values that varied between the given range and results of the FE with the minimum and maximum coefficient of friction were used to compare the analytical solution with the experimental data. Further discussion is provided in the section below.

## Results

**Figure 6:**
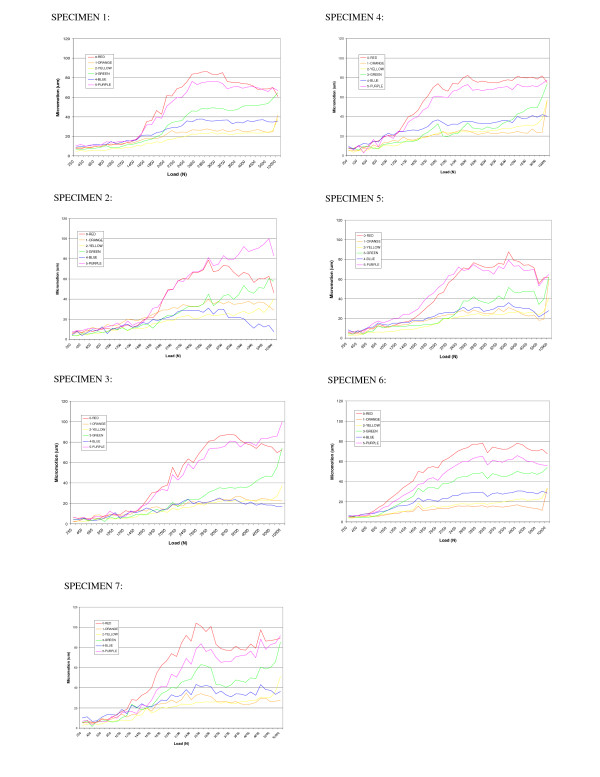
Maximum micromotions achieved for each of the seven liner specimen analyzed.

Figure 6 shows the micromotion measured for each of the seven liner specimens studied. Each graph corresponds to one liner specimen. The six curves in each graph represent the maximum micromotion achieved for each of the six LVDT sensors. The maximum physiological loads experienced in hip are within a range of 1700 to 2200 N. As described in this study, the maximum load applied to each liner specimen during the validation experiment was 10,000 N, which is beyond the attainable force between the acetabular liner and the femoral head in the body. The objective of carrying the experiment beyond 2200 N is to investigate how the liner responds to higher loads and whether there is drastic change in deformation (plastic deformation) where certain peak forces are achieved.

As described previously, a loading profile, similar to the one in the experiment, was used in the FE model. According to the experimental set-up, the micromotion results corresponded to six specific points, where the sensors' tips came in contact with the liner. The corresponding points were identified in the FE model. Because the LVDT sensors only measure displacements along their longitudinal axes, those directions were identified with simple trigonometric calculation in the FE model through superposition of the experimental axes. The micromotions (displacements) were computed in the FE model of the nodes positioned at the reference points, along the identified direction.

**Figure 7:**
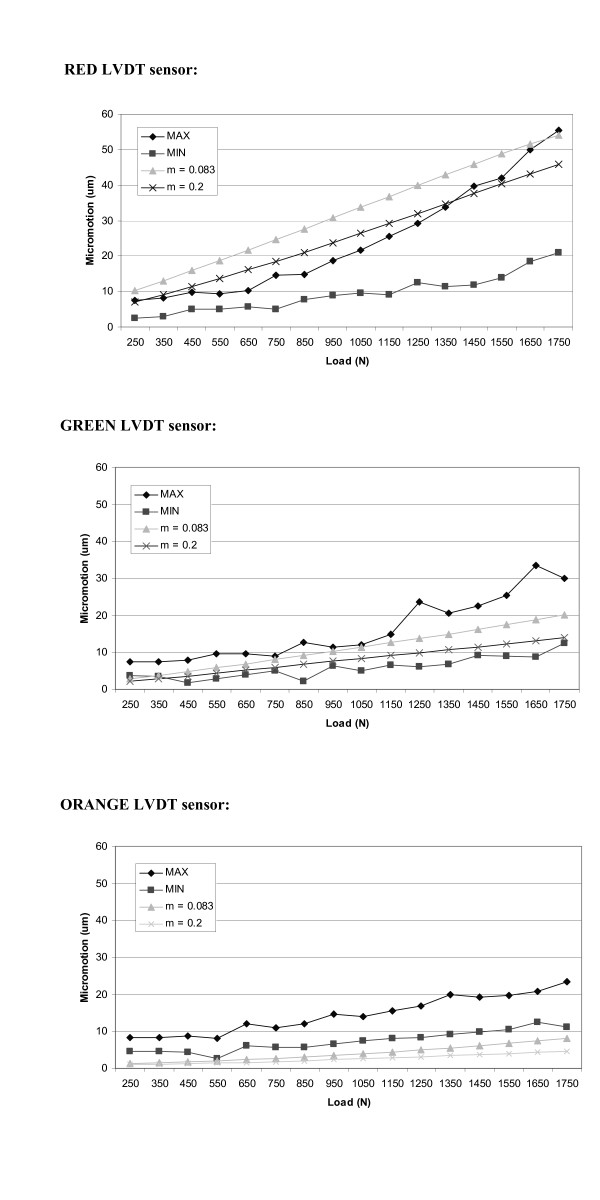
Maximum and minimum micromotions values, among all the specimens analyzed, recorded for each sensor. Comparison with the results obtained with the FE model considering two different friction coefficients.

**Figure 8:**
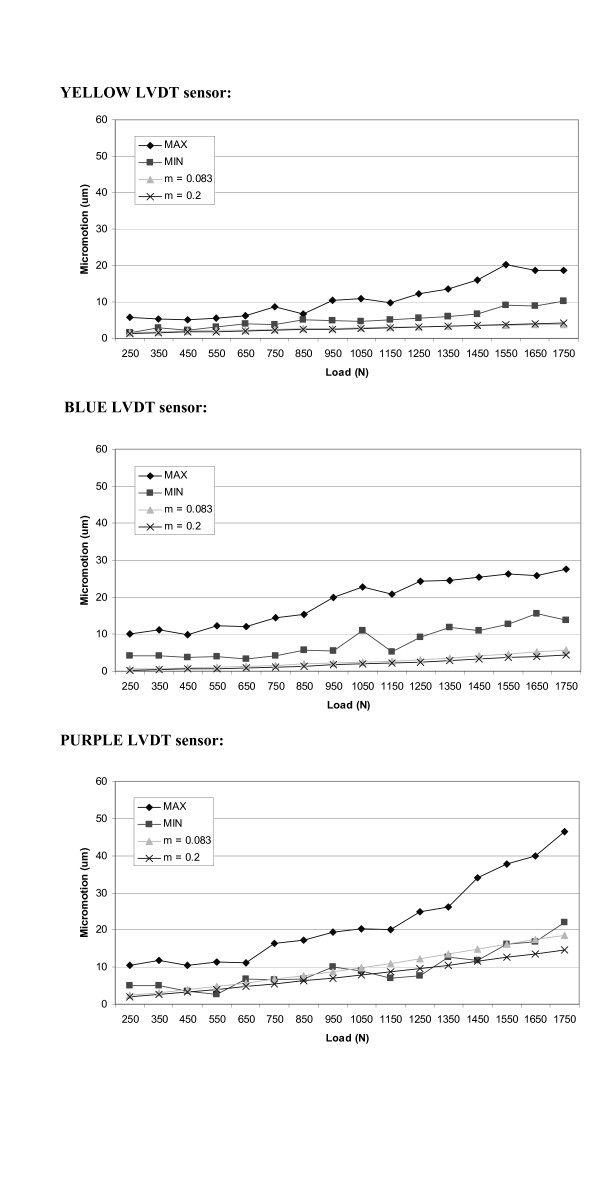
Maximum and minimum micromotions values, among all the specimens analyzed, recorded for each sensor. Comparison with the results obtained with the FE model considering two different friction coefficients.

Figures 7 and 8 indicate the maximum and minimum deformation (micromotion) values, for all the seven liners analyzed, where each sensor was compared to the result obtained with the finite element model. The FE model results are shown for two values of coefficient of friction (μ = 0.2 and μ = 0.083). As expected, the sensors positioned perpendicular to the outer flat surface of the liner recorded the most significant micromotion. This is attributed to the fact that this is the direction along which the liner is less constrained. The three sensors positioned perpendicularly to the side of the liner recorded very little micromotion. This micromotion was produced because of local deformations from the bulging effect of the polyethylene when subjected to compressive loads.

In all the experimental data collected from the seven specimens the result obtained in the FE model, for both friction coefficients, are between the maximum and minimum values obtained in the experiment. This is a first check on the validity of the model and its analytical prediction. Considering the average values for the experiment results (based on the maximum and minimum micromotion values collected for each load), the FE model with the lowest value of showed a closer fit and depicts a more realistic response. When modeling conformity it is important to note that the actual liner and cup do not possess such a characteristic, in fact there are gaps due to the geometrical difference of the two components. These clearances will make the friction between the liner and the cup less pronounced; hence a relaxed value in the FE model is more appropriate. Our validation supports such a scenario.

## Discussion

This study highlights two main points. One the experiment provides an insight into the stability and micromotion of the liner/cup interface. Indeed it provides the means by which we can quantify the micromotion/load interface in relation to the liner and locking mechanism designs. Second these experiments along with the FE model becomes an important tool in identification of weak spots and areas where both stress and wear can be identified. The objective of this research while focused on several specimens the design of the locking mechanism of the liner with respect to the cup is the same. In future studies we intend to perform a much larger study where different locking mechanisms designs can be compared under different loads and moments.

The current investigation has prompted us to suggest areas where we can improve both the FE model as well as learning further how the liners designs affect the results of micromotion and wear. These include: the conformity of the contact surfaces, geometry and tolerances of the CAD model, representation of the liner locking mechanism and material properties. Conformity allows distribution of load more along the equatorial surface of the load and might increase back wear when the material expand and slide along the surface at he interface of the cup/liner. The conformity might also play a significant role in load transfer to the cup and hence changing the micromotion condition at the cup/bone interface. The cup bone interface is a subject of another investigation currently being conducted at our Laboratory.

The results of the validation show that the correlation of the micromotion data given by the FE model and the experimental data depend upon the position of each sensor. The sensors positioned along the flat surface of the liner recorded values that closely matched the model. Among those three sensors, the one positioned further to the axis of application of the load registered the best results. The other two sensors positioned in the flat surface of the liner (red and purple) matched closely the results only in the load ranges between 1500 N and 1750 N. Furthermore, the results obtained for the three sensors positioned perpendicularly to the flat surface of the liner are in the same range of the values obtained from the experiment. Therefore, it is clear that the sensors subjected to higher bulging/deformation showed a slight deviation from those in the FE model. These variations might be a consequence of the assumptions made in selection of polyethylene material. A more precise material model would certainly describe the bulging effects more accurately. For example a more accurate description of the material used in the model would be a piecewise linear isotropic plasticity material [16]. This planned in our future research development.

Another important assumption made in the model is the treatment of the liner's locking tabs. As describe earlier, the locking tabs were simulated constraining all the degrees of freedom of the nodes placed in the same position as the actual locking tabs. This assumption states that the nodes simulating the locking tabs were completely fixed (constrained) to the acetabular shell and may induce high stress concentration areas. In reality those tabs are designed to have significant gap around them when the liner is fully seated. Surely this factor might permit a more even stress distribution in the elements surrounding the constrained nodes of the locking tabs. This might result in a smaller peak stress values at the nodes.

The last hypothesis considered in the present study is concerned with the conformity of the contact areas. Perfect conformity was assumed between liner's backside and acetabular shell inner-surface and between liner's front-side surface and femoral head. In the actual acetabular components certain gaps are allowed between contacting surfaces for both design and manufacturer tolerances. The stress distributions and micromotions achieved in the liner might be different if those gaps were considered and the results obtained might be closer than the one obtained in this study.

## Conclusion

The objective of the present study was to validate a FE model of modular acetabular prosthesis with data collected experimentally. The model is preliminary and gives a reasonable approximation of values of micromotion obtained experimentally. Further work is needed to enhance the model development and improve the accuracy of the model.

## Abbreviations

E (Young Modulus): MPa

Kg (Kilogram): Unit of mass (SI)

m (meter): Unit of Length (SI)

MPa (Mega Pascal): Unit of Pressure (SI)

N (Newton): Unit of Force (SI)

min (minute): Unit of Time equal to 60 seconds.

mm (millimeter): Unit of Length equal to one 10^-3 ^meters.

sec (Second): Unit of Time (SI)

μ (mu): Friction Coefficient (Unit-less)

μm (micron):Unit of Length equal to 10^-6 ^meters

ρ (rho): Symbol for density. Density units Kg/m^3^

ν (nu): Poisson's ratrio (Unit-less)
